# Isoniazid Preventive Therapy and Risk for Resistant Tuberculosis

**DOI:** 10.3201/eid1205.050681

**Published:** 2006-05

**Authors:** Maria Elvira Balcells, Sara L. Thomas, Peter Godfrey-Faussett, Alison D. Grant

**Affiliations:** * London School of Hygiene and Tropical Medicine, London, United Kingdom

**Keywords:** tuberculosis, review, isoniazid, chemoprevention, prophylaxis, drug resistance, research

## Abstract

In the context of tuberculosis (TB) resurgence, isoniazid preventive therapy (IPT) is increasingly promoted, but concerns about the risk for development of isoniazid-resistant tuberculosis may hinder its widespread implementation. We conducted a systematic review of data published since 1951 to assess the effect of primary IPT on the risk for isoniazid-resistant TB. Different definitions of isoniazid resistance were used, which affected summary effect estimates; we report the most consistent results. When all 13 studies (N = 18,095 persons in isoniazid groups and N = 17,985 persons in control groups) were combined, the summary relative risk for resistance was 1.45 (95% confidence interval 0.85–2.47). Results were similar when studies of HIV-uninfected and HIV-infected persons were considered separately. Analyses were limited by small numbers and incomplete testing of isolates, but findings do not exclude an increased risk for isoniazid-resistant TB after IPT. The diagnosis of active TB should be excluded before IPT. Continued surveillance for isoniazid resistance is essential.

## Abstract 2

Preventive therapy may increase risk for drug resistance.

Tuberculosis (TB) has reemerged as a major threat to global public health. Its incidence is rising, particularly in countries with a high HIV prevalence (*1*). HIV-infected persons have an increased risk for reactivated latent TB infection (*2*), of having new TB infection progress rapidly to active disease (*3**,**4*), and of dying during a TB episode (*5*).

Since current TB control methods appear inadequate to prevent the rise in TB incidence among HIV-infected persons in settings with high TB prevalence (*6*), additional measures are required. Studies in the late 1980s and 1990s found that TB "preventive therapy" (treatment of latent TB infection) reduced TB incidence among HIV-infected persons, at least among those with positive tuberculin skin test results (*7*). However, despite recommendations from the World Health Organization (WHO) and the Joint United Nations Programme on HIV/AIDS in 1998 (*8*), TB preventive therapy has not been widely adopted. One obstacle to more widespread use is the concern that using isoniazid monotherapy to treat latent TB infection could promote isoniazid-resistant TB; a literature review in 1970 concluded that, since the introduction of isoniazid in 1952, no evidence existed to support this conclusion (*9*).

Since then, a number of placebo-controlled trials of isoniazid preventive therapy (IPT) have been conducted, mostly among HIV-infected persons. We carried out a systematic review of studies (in both the pre-HIV and the HIV era) that compared those who received IPT to an untreated group and reported data on resistance to isoniazid, aiming to assess the effect of primary IPT on the risk of developing isoniazid-resistant TB.

## Methods

### Identification and Selection of Studies

We searched 5 electronic databases (PubMed, Embase, Popline, National Library of Medicine Gateway, Cochrane Library) to identify studies of IPT published in English, French, or Spanish from 1951 to October 2003. Thesaurus and free-text terms were used in various combinations, depending on the requirements of each database (details available on request). We also searched by hand the reference lists in all identified publications and recent systematic reviews (*7**,**10**–**12*).

We reviewed the full text of all studies evaluating the effectiveness of primary IPT (given to persons with no history of TB), applying the following inclusion criteria: 1) compared incidence of TB in persons receiving isoniazid monotherapy versus those receiving no preventive therapy; 2) randomized controlled trial (RCT) or cohort study designs; and 3) results of susceptibility testing of positive cultures presented for both isoniazid and control groups, so the proportion of resistant strains could be ascertained in each group. We excluded studies conducted only in children (among whom microbiologic confirmation is less common), studies of secondary preventive therapy, and studies, or subgroups within studies, of persons with "recently active disease," many of whom had previously received isoniazid.

Data were extracted in duplicate by 2 investigators independently, using a standardized data-collection form. Data included study details (study population and size, design, intervention drug regimen, outcomes recorded) and quality measures (e.g., generation and concealment of allocation sequences, blinding, duration of and loss to follow-up).

### Statistical Analysis

We estimated the incidence of TB caused by isoniazid-resistant strains separately for the isoniazid and control group of each study by dividing the number of persons with isoniazid-resistant TB by the total number of persons in that group. We chose the incidence of isoniazid-resistant TB in preference to the proportion of culture-positive TB cases that were isoniazid resistant because incidence better represents the impact (and risk for transmission) of resistant disease at the population level. Also, comparison of the proportion of resistant isolates between groups is complicated if the study population includes persons who have latent TB infection with an isoniazid-resistant organism. In the group receiving isoniazid, preventive therapy will decrease the number of reactivated TB cases attributable to isoniazid-susceptible strains but will have less effect on resistant strains, which will increase the proportion of resistant strains among subsequent cases of active TB. As a result, the proportion of isoniazid-resistant active TB cases will be higher in the isoniazid group than in the control group, even if isoniazid does not promote new resistance.

The analysis involved a number of assumptions, summarized in Table 1. In studies in which not all TB patients underwent resistance testing, we assumed that isolates tested were a random sample of all TB cases and multiplied the total number of TB cases by the proportion of isoniazid-resistant cases in the sample to estimate the total number of isoniazid-resistant cases. For example, if 1,000 persons were randomly assigned to isoniazid therapy, active TB developed in 50, 40 of these were tested, and 8 (20%) of 40 had isoniazid-resistant isolates, we then estimated a total of 10 (50 × 0.2) resistant TB cases and an incidence of isoniazid-resistant TB of 10 per 1,000 persons.

**Table 1:** Assumptions underlying the analysis

Assumption	Comment
When a sample of culture positive isolates underwent resistance testing, this was a random sample of all cases.	Additional variation incurred by sampling tuberculosis (TB) cases for resistance was incorporated into 95% confidence interval estimates and thus the weighting of studies in meta-analyses.
	Differential ascertainment of resistance is unlikely because most of the included studies were double-blinded and (for studies in which information was available) similar proportions of culture-positive TB cases from each group were tested.
Latent infection with isoniazid-resistant TB was equally distributed between comparison groups.	12 of 13 studies were comparisons of randomized groups; any latent infection with a resistant organism would likely be equally distributed between comparison groups. Any imbalance due to random error would be bidirectional and so would result in summary estimate of relative risk tending towards 1 (i.e., being underestimated).
Risk for isoniazid-resistant TB resulting from recent infection was equally distributed between comparison groups.	Similarly, any new infection with an isoniazid-resistant organism would likely be equally distributed between randomized groups. Any imbalance would similarly result in summary estimate of relative risk being underestimated.

Relative risks (RR) for resistant TB in the isoniazid group compared to the control group were calculated for each study. The extra variation incurred by sampling isolates for resistance was incorporated into the 95% confidence intervals (CIs) of each RR. The RR could be written as the product of 2 ratios (the ratio of TB incidence in exposed/unexposed multiplied by the ratio of the proportion of resistant cases in the sample tested for the exposed/unexposed). Thus, the log RR could be expressed as the sum of the logs of these ratios, and the variance of the log RR could be calculated by a double application of a standard formula (details available on request). When no resistant cases were found in 1 of the 2 groups, we added 0.5 to the numerator and denominator of both groups when estimating the risk, and 0.1 to the numerators and denominators when calculating the variance of the ratio of proportions (*13*).

Tests of between-study heterogeneity were performed, and meta-analyses were carried out to derive summary RRs, by using a random-effects model when evidence of heterogeneity was found (*14*). In the meta-analysis, we first considered all studies as a single group, then considered separately studies from the pre-HIV era and studies of HIV-infected persons; we hypothesized that HIV-infected persons could be at higher risk of having resistance develop. When latent TB infection is treated, few organisms are exposed to the drug (*15*). The risk for selection pressure favoring a drug-resistant organism is therefore low (*16*) unless persons have undiagnosed active TB and thus inadvertently receive monotherapy for active disease. Active TB may be more difficult to detect among HIV-infected persons, which could lead to a higher risk for undiagnosed active disease.

Sensitivity analyses primarily consisted of excluding from meta-analyses studies a) that had zero resistant cases in a group and b) that were not RCTs. Publication bias was investigated by using funnel plots and adjusted rank correlation tests (*17*). All analyses were carried out in Stata version 8.0 (Stata Corp., College Station, TX, USA).

## Results

We identified 19 studies comparing primary IPT with no treatment that reported isoniazid resistance among adults (*9**,**18**–**35*). Of the 11 studies from the pre-HIV era, 4 (*23**–**26*) were excluded because resistance data from the control group were incomplete or not reported. In 2 studies (*9**,**19*), we excluded subgroups of persons with previously active disease, for which many had received isoniazid. Of the 8 studies among HIV-infected persons, 2 (*28**,**29*) were excluded because the total number of isolates tested in the relevant groups could not be determined. For 1 study (*33*), unpublished resistance data were obtained from the authors (P. Godfrey-Faussett, pers. comm.).

### Characteristics of Included Studies

Thirteen studies were included in the analysis (Tables 2, 3), 12 RCTs and 1 retrospective cohort study. The 7 pre-HIV era studies (N = 32,179) were mostly conducted in the late 1950s or early 1960s in populations of persons with radiologically-inactive TB lesions (*9**,**19**,**22*), persons in communities with high TB incidence (*20**,**21*), and household contacts of TB cases (*18*); 1 study was of persons with silicosis in Hong Kong in the 1980s (*27*). Study population size ranged from 225 to 15,751 patients. In most studies, isoniazid 300 mg (or 5 mg/kg) was given daily, although in the Greenland study (*20*), 400 mg was given on 2 consecutive days each week. Duration of treatment was 24 weeks to 2 years. All 6 studies among HIV-infected persons (N = 3,901) recruited participants from HIV clinics or voluntary counseling and testing centers. Study population size was 121–1,718. RCTs administered isoniazid for 6 months at 300 mg daily (*30**,**31**,**34**,**35*) or 900 mg twice weekly (*33*); in the cohort study, an unspecified dose was given for 9 to 12 months (*32*).

**Table 2:** Studies comparing isoniazid treatment with no treatment in HIV-uninfected populations*

Author, country, dates	Population	Intervention/comparison; blinding	Enrolled (n) INH/control	Follow-up; loss to follow-up; overall or INH vs. control	TB cases: culture positive/total (%)		Definition of INH resistance	Resistant cases/total tested (% culture positive tested)		Risk for resistant TB/1,000		
					INH	Controls		INH	Controls	INH	Controls	RR (95% CI)
Ferebee, USA, 1957–NS (*18*)	Household contacts of TB patients	12 mo INH, 4–7 mg/kg/day/placebo; double blind	7,755/7,996	<10 y; 5.2% vs. 4.9% during Rx	NS/86	NS/215	>50 colonies growth in 0.2 μg/mL INH	2/10 NS	2/31 NS	2.22	1.73	1.28 (0.20–8.07)
Katz, USA, 1958–1964 (*19*)	Mental hospital patients with inactive lesions	2 y INH, 300 mg daily/no treatment; not blind	118/107	<4 y post-Rx; 30.6% overall†	NS/9	NS/10	Resistance to >0.25 γ INH	1/1 NS	2/5 NS	76.27	37.38	2.04 (0.52–8.08)
Horwitz, Greenland, 1956–1963 (*20*)	76 villages	2 × 13 wk INH, 400 mg twice weekly/0.1 mg INH; double blind	4,174/3,907	6 y; NS	123/238 (51.7)	186/323 (57.6)	(a) > 1 colony at >0.32 μg/mL INH	(a) 2/46	(a) 5/66	(a) 2.48	(a) 6.26	(a) 0.40 (0.08–1.97)
							(b) Equal to control tube at >0.32 μg/mL INH	(b) 2/46 (37)	(b) 1/66 (36)	(b) 2.48	(b) 1.25	(b) 1.98 (0.18–21.31)
Comstock, USA (Alaska), 1957–1964 (*21*)	Residents of 28 villages and 2 boarding schools	12 mo INH, 300 mg§ daily/placebo; double blind	3,047/3,017	Med.: 69.3 mo (range 43–76 mo); 5.3% observed for <40 mo	NS/58	NS/141	NS	4/20 NS	1/50 NS	3.81	0.93	4.07 (0.47–34.98)
Ferebee, USA, 1960–1967 (*9*)	Persons with inactive lesions	12 mo INH, 5 m g/kg/day/placebo; NS	701/714	5 y; 2.2% by 1967	NS/18	NS/49	>50 colonies growth in 0.2 μg/mL INH	2/5 NS	2/25 NS	10.27	5.49	1.87 (0.31–11.19)
Pamra, India, 1958–1968 (*22*)	X-ray screening attendees with inactive TB	12 mo INH, 3–4 mg/kg/day/placebo; NS	139/178	<5 y post-Rx; 8.6% vs. 11.2%	10/18 (55.6)	57/76 (75)	Growth on 1 μg/mL INH	3/9 (90)	6/52 (91)	43.17	49.27	0.88 (0.24–3.15)
Hong Kong Chest Service, Hong Kong, 1981–1987 (*27*)	Men with silicosis	24 wk INH, 300 mg daily/placebo;double blind	167/159	2–5 y; 15.8% at 5 y	19/25 (76)	29/36 (80.6)	>20 colonies in >1 culture at >0.2 mg/L INH	5/19 (100)	4/28 (97)	39.39	32.35	1.22 (0.34–4.32)

*INH, isoniazid; TB, tuberculosis; RR, relative risk; CI, confidence interval; NS, not stated; med., median; Rx, treatment. †(a), definition of resistance as >1 colony growth at >0.32 μg/mL INH. ‡(b), definition of resistance as growth equal to control tube at >0.32 μg/mL INH. §Children were given 5 mg/kg/day INH.

**Table 3:** Studies comparing isoniazid treatment with no treatment in HIV-infected populations*

Author, country, dates	Population	Intervention/ comparison; blinding	Enrolled (n) INH/control	Follow up; loss to follow-up; overall or INH vs. control	TB cases: culture positive/total (%)		Definition of INH resistance	Resistant cases/total tested (% culture positive tested)		Risk for resistant TB/1,000		
					INH	Controls		INH	Controls	INH	Controls	RR (95% CI)
Randomized controlled trials												
Gordin, USA, 1991–1996 (*30*)	Clinic attendees; med. CD4 233/247	6 mo INH 300 mg daily vs. placebo; double blind	260/257	34 mo/33 mo; 6.2% vs. 7%	NS/3	NS/6	NS	0/3 (NS)	0/5 (NS)	1.92†	1.94†	0.99 (0.06–6,298.19)
Hawken, Kenya, 1992–1997 (*31*)	Clinic or VCT attendees; med. CD4 321.5/346	6 mo INH 300 mg daily/placebo; double blind	342/342	Med. 1.83 y (range 0–3.41); 32% vs. 27.3% not seen in final 6 m	19/25 (76)	22/23 (95.7)	Growth on 0.2 μg/mL INH >1% growth on control medium	2/17 (90)	0/21 (96)	10.05†	1.46†	6.88 (0.01–3,882.85)
Mwinga, Zambia, 1992–1996 (*33*)	VCT attendees	6 mo INH 900 mg twice weekly/placebo; double blind	350/352	Med. 1.8 y; 32.4% vs. 30.3% not seen in final 6 m	NS/27	NS/44	NS	0/3 (NS)	1/5 (NS)	1.43†	26.38†	0.05 (0.00–30.47)
Johnson, Uganda, 1993–NS (*34*)	Clinic or counseling attendees	6 mo INH 300 mg daily/placebo; partially double blind‡	931/787	Mean 2 y/1.6 y (PPD+/anergic); 16.1% vs. 30.6%	36/51 (70.6)	46/64 (71.9)	Growth on 0.1 μg/mL INH (BACTEC radiometric method) >1% growth on control medium	5/20 (56)	1/24 (52)	13.69	3.39	4.04 (0.50–32.80)
Rivero, Spain, 1994–2000 (*35*)	Clinic attendees; med. CD4 193/215	6 mo INH 300 mg daily/no treatment; not blind	82/77	24 mo; 26.8% vs. 7.8%	3/3 (100)	4/4 (100)	NS	3/3 (100)	4/4 (100)	36.59	51.95	0.70 (0.16–3.05)
Cohort study												
Moreno, Spain, 1985–1994 (*32*)	Clinic attendees; med. CD4 689/648	9–12 mo INH (dose NS)/no treatment; not blind	29/92	89 mo vs. 60 mo; NS	3/3 (100)	39/43 (90.7)	Growth on 0.2 μg/mL INH >1% growth on control medium	2/2 (67)	0/12 (31)	118.64†	5.41†	21.95 (0.04–11,582.31)

*INH, isoniazid; TB, tuberculosis; RR, relative risk; CI, confidence interval; med., median; NS, not stated; Rx, treatment; VCT, voluntary counseling and testing; PPD, purified protein derivative. †Calculated by adding 0.5 to numerator and denominator of both groups. ‡Unclear whether isoniazid and placebo group received the same number of tablets.

We could assess the method of assigning the treatment allocation in 5 of the 12 RCTs: 2 studies (*31**,**33*) used computer-generated random numbers, 2 (*20**,**21*) used random number tables, and 1 (*19*) assigned by odd or even hospital number. Three RCTs reported that the treatment was concealed: 2 used sealed envelopes (*33**,**34*), and 1 used numbered packages containing isoniazid or matching placebo (*27*). Eight RCTs were double-blinded (*18**,**20**,**21**,**27**,**30**,**31**,**33**,**34*), although in 1 study, isoniazid and placebo groups may have received different numbers of tablets (*34*); 2 were not blinded (*19**,**35*), and 2 did not report blinding (*9**,**22*). Loss to follow-up was reported in 11 studies: in 6, this loss was <20% in both groups (Tables 2, 3).

### Tuberculosis Cases and the Proportion of Isoniazid-resistant Isolates

The total number of TB cases within a study ranged from 7 to 561. In all studies combined, 564 TB cases occurred among persons who received isoniazid, and 1,034 occurred among controls. In the 7 studies that reported this information, 55%–100% of TB cases were sputum-culture positive (*20**,**22**,**27**,**31**,**32**,**34**,**35*). In 4 of these studies, >90% of culture-positive isolates underwent resistance testing (*22**,**27**,**31**,**35*). In total, 158 persons in the isoniazid groups and 328 in control groups had isolates tested for resistance to isoniazid. Definitions of isoniazid resistance varied, and the proportion of tested isolates that were resistant ranged from 0% to 100% (Tables 2, 3). A total of 31 resistant isolates were obtained from the isoniazid groups and 28 or 24 (depending on the definition of resistance) from control groups. Of the 6 studies among HIV-infected persons, 1 found no resistant isolates in the isoniazid group (*33*), 2 found no resistant isolates in the control group (*31**,**32*), and 1 found no resistance in either group (*30*) (Table 3).

**Figure 1:**
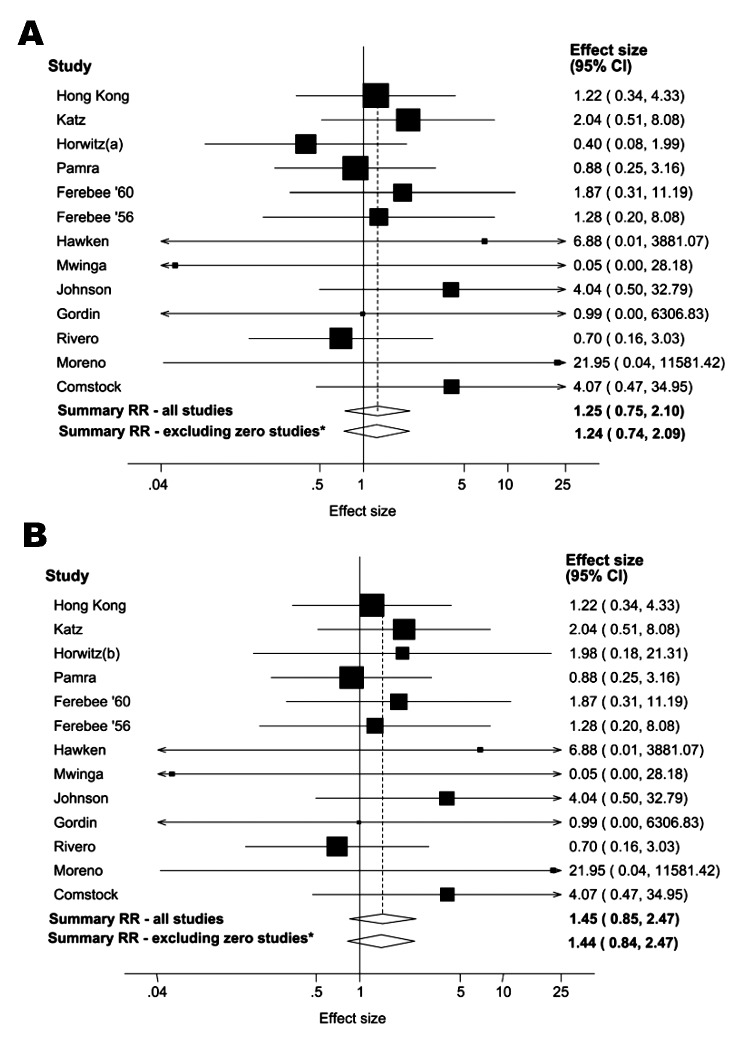
Relative risk (RR) for isoniazid resistance associated with isoniazid preventive therapy in 13 studies. A) Using definition (a) of resistance for the Greenland study (*20*). B) Using definition (b) of resistance for the Greenland study. *Excluding the 4 studies with no resistant cases in 1 or both of the 2 groups. The squares and horizontal lines represent the relative risk (RR) and 95% confidence intervals (CIs) for each study. The diamonds represent the summary RR and 95% CIs.

In 8 of the 12 studies in which a single definition of resistance was used, the point estimate of RR for isoniazid resistance in the isoniazid group compared to that of controls was >1, although this result was not statistically significant in any study (Tables 2, 3). Two alternative (and substantially different) definitions of resistance were used in the Greenland study, which resulted in different estimates of the effect of IPT on isoniazid resistance (Table 2). We therefore conducted 2 analyses, using each definition of resistance for this study. By using definition (a) from the Greenland study, the summary RR for all 13 studies combined was 1.25 (95% CI 0.75–2.10) in either a random or fixed effects model (Figure 1A) with little evidence of heterogeneity (P_het_ = 0.789). By using definition (b) from the Greenland study, the summary RR was 1.45 (95% CI 0.85–2.47, Figure 1B), again with little evidence of heterogeneity (P_het_ = 0.923). Summary estimates were virtually unaltered when analyses were restricted to RCTs without zero cells (Figure 1). We also excluded the Greenland study from the meta-analysis to assess its overall effect on the summary estimate. The summary RR using the remaining 12 studies was similar to that obtained by using definition (b) for resistance (RR 1.43, 95% CI 0.83–2.46).

**Figure 2:**
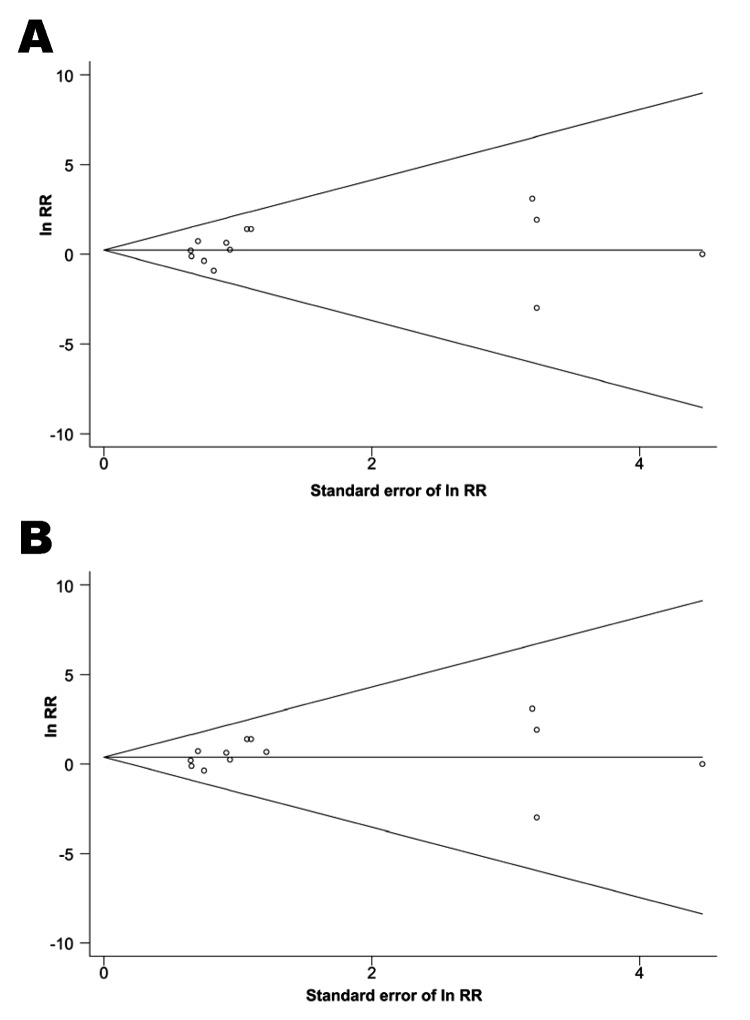
Funnel plots to detect publication bias for studies reporting the effect of isoniazid preventive therapy on risk for isoniazid-resistant tuberculosis. The log relative risk (RR) for each study is plotted against the standard error of the natural log (ln) of the RR. The horizontal line indicates the (log) summary RR, and guidelines to assist in visualizing the funnel are plotted at the 95% pseudoconfidence limits about the summary RR estimate. A) Using definition (a) of resistance for the Greenland study (*20*); B) using definition (b) of resistance for the Greenland study.

Among the 7 studies from the pre-HIV era, the summary RR for isoniazid resistance was 1.24 (95% CI 0.69–2.21) when the definition (a) from the Greenland study was used and 1.50 (95% CI 0.82–2.73) with definition (b). The summary RR was 1.30 (95% CI 0.42–4.02) for the 6 studies of HIV-infected persons. Little evidence of between-study heterogeneity was found in any of these analyses (P_het_ >0.5 for all). When meta-analysis of the studies of HIV-infected persons was restricted to the 2 RCTs without zero cells (*34**,**35*), the summary RR rose slightly to 1.42 (95% CI 0.26–7.69) in a random-effects model, with slightly stronger evidence of heterogeneity (P_het_ = 0.179). Funnel plots (Figure 2) suggested little evidence of publication bias (p = 0.625 and p = 0.542 by using definition [a] and definition [b], respectively, for the Greenland study).

## Discussion

Our summary RR for isoniazid-resistant TB after IPT is not statistically significant, but the point estimate and upper boundary of the 95% CI are consistent with an increased risk. Our review highlights the limitations of existing data; however, since further individually randomized, controlled trials of IPT would be inappropriate, additional data of this type are unlikely to become available.

The numbers of TB cases in the individual studies were often small, and in 4 studies, no resistant TB cases occurred in at least 1 of the comparison groups. Comparison of summary estimates with and without these 4 studies suggests that adding a small number to the numerators and denominators so they could contribute to summary estimates did not in itself affect the result. The 95% CIs for RRs in these studies were very wide, and so their contribution to the summary RR estimate was limited.

The summary estimate of effect was similar in HIV-infected and HIV-uninfected persons. Screening for active TB before enrollment could have been more rigorous in studies among HIV-infected persons; the screening procedures were not always clearly described.

The proportion of positive cultures tested for resistance varied from 37% to 100%; why all isolates were not tested was not clear. The most important assumption made in the analysis was that the proportion of resistant cases among the isolates tested was representative of all TB cases in that group. If investigators were not blinded to the treatment allocation, and if persons receiving isoniazid were more likely to have positive cultures tested for resistance, ascertainment of resistance in the isoniazid group could have increased, and thus RR could have been overestimated. However, in 10 of the 13 studies, a placebo was used; 8 studies specified that the trial was double blinded, and (for studies for which information was available) similar proportions of culture-positive TB cases were tested from each group. Therefore, differential ascertainment of resistance is unlikely. Our estimate of the total number of isoniazid-resistant cases disregarded whether case-patients were sputum-culture positive. Persons with isoniazid-resistant isolates that are sputum-culture negative are less likely to transmit disease and present less of a public health concern. This situation is unlikely to affect our estimate of the effect of isoniazid on the incidence of resistant disease, but our estimate may exaggerate the public health risk.

Study quality and review methods may have affected the results in other ways. For example, inadequate random assignment of HIV-infected persons could result in more advanced immunosuppression among those in the isoniazid group and thus a higher probability of resistance. However, when reported, the method of randomization in trials of HIV-infected persons appeared adequate. Differences in loss to follow-up between comparison groups could affect results if those who were lost to follow-up had a different probability of resistance than those not lost. In 6 of the 11 RCTs with information, <20% were lost to follow-up in both groups, but the loss was noticeably higher in the isoniazid group than for controls in 2 studies of HIV-infected persons (*34**,**35*). Publication bias could affect the results if studies finding increased resistance among persons receiving isoniazid were more likely to be published. However, the aim of all the studies was to investigate effectiveness of IPT, not to ascertain development of resistance, and our analyses suggest that publication bias did not affect the summary estimate.

The methods used to test for isoniazid resistance are now relatively standardized and based on the proportion method in which resistance is defined as growth on medium containing 0.2 μg/mL isoniazid that exceeds 1% of the growth on control medium (*36*). In older studies, methods were less standardized and were based on absolute numbers of colonies growing on media with various concentrations of antituberculous drugs. In the Greenland study, results for resistance were presented by using 2 divergent definitions (neither corresponding to modern methods), and these gave quite different estimates of effect. Definition (a) is likely to have led to an overestimation of resistance in both groups; definition (b) is likely to have led to an underestimation of resistance in both groups. When this study was excluded from the analysis, the summary estimate was similar to that using definition (b), which suggests that the estimates using definition (a) were more anomalous.

Studies using DNA fingerprinting illustrate that in settings with a high prevalence of TB, newly acquired infection is an important cause of active TB (*37**,**38*). Thus, isoniazid-resistant TB may be newly acquired rather than attributable to any previous IPT. However, any such effect should be equally distributed between randomized groups (Table 1).

IPT is a safe, low-cost intervention that has the potential to reduce illness and death caused by TB, especially among HIV-infected persons. The main cause of antituberculous drug resistance is inadequate treatment of active TB. Therefore, any risk for a small increase in the incidence of isoniazid resistance attributable to wider use of IPT needs to be weighed against its benefit in reducing TB incidence.

If IPT does increase the risk for isoniazid-resistant TB, one might argue that combination regimens should be used. Combination regimens have similar efficacy to isoniazid alone among HIV-infected persons and are shorter, but these regimens generally have more adverse effects (*7**,**39*), are more expensive, and risk promoting resistance to rifampin. We did not compare the risk for antituberculous drug resistance with IPT versus combination regimens.

Our review highlights the paucity of available data and does not exclude an increased risk for isoniazid-resistant TB after IPT. IPT substantially reduces the risk for active TB disease in persons whose tuberculin skin test is positive, and we support the expansion of its use, in line with recent recommendations from the HIV/TB working group of the Stop TB partnership (*40*). If the main reason for the development of resistance among persons receiving IPT is failure to diagnose active TB, our results underscore the need for effective diagnostic strategies and tests. In accordance with WHO policy, ongoing surveillance for isoniazid resistance is required among populations in which this intervention is widely implemented.
